# The MicroRNA miR-454 and the mediator complex component MED12 are regulators of the androgen receptor pathway in prostate cancer

**DOI:** 10.1038/s41598-025-95250-0

**Published:** 2025-03-25

**Authors:** Juan Guzman, Martin Hart, Katrin Weigelt, Angela Neumann, Achim Aigner, Chiara Andolfi, Florian Handle, Stefanie Rheinheimer, Ulrike Fischer, Uta D. Immel, Verena Lieb, Eckart Meese, Zoran Culig, Bernd Wullich, Helge Taubert, Sven Wach

**Affiliations:** 1 Department of Urology and Pediatric Urology, Uniklinikum Erlangen, Friedrich-Alexander-Universität Erlangen-Nürnberg, 91054 Erlangen, Germany; 2 Comprehensive Cancer Center Erlangen-EMN (CCC ER-EMN), 91054 Erlangen, Germany; 3 Center of Human and Molecular Biology (ZHMB), Institute of Human Genetics, Saarland University (USAAR), 66421 Homburg, Germany; 4 Rudolf-Boehm-Institute for Pharmacology and Toxicology, Clinical Pharmacology, University of Leipzig, Leipzig, Germany; 5 Department of Urology, Division of Experimental Urology, Medical University of Innsbruck, 6020 Innsbruck, Austria; 6 Institute of Pathology, Neuropathology & Molecular Pathology, Medical University of Innsbruck, 6020 Innsbruck, Austria; 7 Institute of Human Genetics, Saarland University (USAAR), 66421 Homburg, Germany; 8 Institute of Legal Medicine, Johannes Gutenberg University Medical Center, 55131 Mainz, Germany; 9 Bavarian Cancer Research Center (BZKF), 91054 Erlangen, Germany

**Keywords:** Prostate cancer, MiRNA, MED12, Cell viability, Protein expression, Androgen receptor, Androgen receptor splice variant 7, c-Myc, Cancer, Cell biology, Genetics, Molecular biology, Biomarkers, Molecular medicine, Risk factors, Urology

## Abstract

Prostate cancer that is resistant to anti-androgen treatment, such as enzalutamide, represents a therapeutic challenge. To study their molecular and functional features, the enzalutamide-resistant PCa cell lines LNCaP Abl EnzR and DuCaP EnzR constitute valuable in vitro models. In this work, we explored two different strategies for reducing AR/AR-V7/c-Myc. MED12 knockdown decreased the protein expression of AR, AR-V7 and c-Myc. Similarly, we identified AR and AR-V7 as targets of miR-454-3p. Concomitantly, the transfection of synthetic miR-454-3p reduced the protein expression of AR in both EnzR cell lines and that of c-Myc and AR-V7 in the DuCaP EnzR cell line without affecting MED12. Despite these similar molecular effects, differences were observed at the cellular level, with siMED12, but not miR-454, reducing cell viability, and no additive effects upon double treatment were observed. Taken together, the results of our study suggest MED12 as a potential target for future PCa treatment in conjunction with enzalutamide resistance. Furthermore, miR-454-3p, which directly targets AR and AR-V7 and indirectly influences c-Myc protein expression, reveals new molecular mechanisms in PCa biology.

**Supplementary Information:**

The online version contains supplementary material available at 10.1038/s41598-025-95250-0.

## Introduction

With an incidence of more than 1.46 million new cases per year worldwide, prostate cancer (PCa) is the most common malignant tumor in elderly men^1^. In its early localized stages, PCa is usually treated with curative intent by local therapy such as radical prostatectomy, external beam radiation therapy or brachytherapy. PCa exhibits a remarkable dependency to androgen receptor (AR) signaling in all stages of disease. Therefore, androgen deprivation therapy (ADT) which disrupt AR signaling by surgical or chemical castration is the next step in PCa therapy. But eventually the progression towards a hormone refractory or castration resistant stage (CRPC) or its metastatic form (mCRPC) occurs, what is the major cause of morbidity and mortality. The mechanisms, by which this resistance may be acquired, are diverse and include gene amplification^2^, pathway hyper-sensitization, androgen receptor gene mutation [reviewed in 3] or the generation of AR splice variants^4,5^. The AR variant 7 (AR-V7) is the most studied PCa relevant AR splice variant which has been correlated with treatment resistance^6,7^. There is still an urgent need to find new therapy options for CRPC/mCRPC.

The Mediator (MED) complex is involved in transcription initiation, elongation, long-range enhancer–promoter interactions and chromatin architecture^8–11^. The MED complex has a very ancient eukaryotic origin^12^. A major function of the MED complex is to relay regulatory signals from DNA-bound transcription factors directly to the RNA Pol II enzyme^11,13^. The MED complex is composed of a core mediator and a kinase module. The kinase module comprises cyclin-dependent kinase 8 (CDK8), cyclin C (CCNC), MED13 and MED12^10^.

The MED complex has been implicated in genomic and nongenomic signaling in cancer^14^. The expression and tumor biological role of MED subunits in prostate cancer (PCa), including CRPC, have been intensely investigated in the last 10 years. Previous studies focused mainly on the subunit MED1 as a transcriptional activator of AR^15^ and a mediator of AR splice variant (ARv567es)-induced gene expression^16^. MED1 overexpression is proposed to have implications in prostate oncogenesis^17^, and activated ERK and AKT signaling drive MED1 overexpression in PCa^18^. A mutation in MED12 has been reported to occur in approximately 5% of PCa cases^19^; however, in our own PCa patient cohort, we were unable to detect this particular mutation^20^. Ten years ago, MED12 was shown to be expressed at increasing levels from androgen-sensitive to local recurrent CRPC and further to distant metastatic CRPC^21^. Nuclear overexpression of MED12 was detected in 40% of patients with distant metastatic CRPC and 21% of patients with locally recurrent CRPC, whereas it was detected in fewer than 11% of patients with androgen-sensitive PCa. Benign prostatic tissue never displays MED12 overexpression^21^. Although the role of MED12 in tumorigenesis and for cancer therapeutics has been recognized^22^, the pivotal yet largely unidentified role of MED12 in CRPC requires further characterization.

We recently showed that MED12 knockdown modulates androgen receptor transcriptional activity and enzalutamide resistance in prostate cancer^23^. MED12 knockdown reduced cell growth in different PCa cell lines and their cognate 3D spheroids. In addition, MED12 knockdown significantly inhibited c-Myc protein expression and signaling through the c-Myc pathway. Consistently, it inhibited AR transcriptional activity, PSA secretion, AR target gene expression and AR-V7 expression in 22Rv1 cells. In combination with enzalutamide, MED12 inhibition additively decreased AR activity in both LNCaP and 22Rv1 cells^23^.

MicroRNAs (miRNAs) are small noncoding RNAs that exert important posttranscriptional control mechanisms. They have been described as tumor suppressors or oncogenes in different cancers, including prostate cancer^24–26^. Major roles of miRNAs in PCa development and progression have been reported by us and others [reviewed in 27]^28–35^, Two miRNA/target prediction tools (StarmiR and miRTarBase) predict that miR-454-3p targets MED12. Interestingly, another miRNA/target prediction tool (miRDB) predicts that miR-454-3p targets the androgen receptor (AR). However, the androgen receptor has not yet been validated as a regulatory target of miR-454-3p.

miR-454 has already been reported as a tumor suppressive miRNA in acute myeloid leukemia, ovarian cancer, non-small cell lung cancer, neuroblastoma and bladder cancer^36–40^. However, for other tumor entities, miR-454 has been described as an oncogenic miRNA, e.g., in hepatocellular carcinoma, vulvar cancer, renal cell carcinoma and breast cancer^41–46^. Meanwhile, in other tumor entities, such as gliomas^47,48^, and in prostate cancer, the role of miR-454 is still ambiguous, as it has been described as both^45^ oncogenic and tumor suppressive. For prostate cancer patients, a decreased miR-454 level in the blood in comparison with healthy individuals has been reported, which suggests a tumor suppressive role^49^, whereas other authors have reported an oncogenic function, e.g., higher miR-454 expression in recurrent and metastatic PCa than in primary PCa^50^, suppression of miR-454 inhibiting the proliferation and invasion of PCa cells via Wnt-β-catenin-NDRG2 signaling^51^, miR-454 promoting PCa metastasis [reviewed in 52], and miR-454 significantly upregulated in PCa compared with adjacent nonmalignant tissue^53^.

In our study, we investigated (i) whether miR-454-3p is able to regulate MED12 and/or AR expression, (ii) whether the inhibition of MED12 by siRNA or the overexpression of miR-454-3p has a functional effect on the viability of EnzR PCa cell lines and (iii) the effects of siMED12 and miR-454-3p on the expression of selected proteins (MED12, AR, AR-V7, and c-Myc) in EnzR PCa cell lines.

## Materials and methods

### Cell lines

All the cell lines were established in the lab of Dr. Zoran Culig, Medical University of Innsbruck. The identity of the cell lines was verified via STR profiling in June 2024, and all the cells were regularly tested for mycoplasma infection.

LNCaP cells, derived from a patient with lymph node metastasis, have a mutated AR containing the T878A amino acid change^54–56^. LNCaP Abl has been generated by long-term androgen ablation from LNCaP cells^57,58^. In addition, LNCaP cells express the AR-V7 splice variant at the mRNA level but not at the protein level^59^. LNCaP Abl EnzR cells have comparable levels of AR and AR-V7 to those of LNCaP Abl cells^58^.

DuCaP cells, derived from a human dura metastasis xenograft, have approximately 80 copies of AR, increasing the expression of AR at the RNA and protein levels^58,60^. In addition, DuCaP cells express the AR-V7 splice variant at the mRNA and protein levels^58^. Compared with DuCaP cells, DuCaP EnzR cells presented further increases in AR mRNA and AR protein levels, which was not attributed to additional AR gene amplification^58^.

The EnzR PCa cell lines LNCaP Abl EnzR and DuCaP EnzR were cultivated in the presence of enzalutamide (LNCaP Abl EnzR: 13 µM; DuCaP EnzR: 8 µM). LNCaP Abl EnzR cells were cultivated in RPMI 1640 media supplemented with 10% charcoal-stripped FBS (HyClone, GE Healthcare, USA), 1 nmol/l L-glutamine, 100 U/ml penicillin and 100 µg/ml streptomycin (PAN-Biotech GmbH, Aidenbach, Germany). DuCaP EnzR cells were grown in RPMI 1640 media supplemented with 10% fetal bovine serum (FBS; PAA laboratories, Coelbe, Germany), 1 nmol/l L-glutamine, 100 U/ml penicillin and 100 µg/ml streptomycin (PAN-Biotech). The cells were cultivated at 37 °C in 5% CO_2_.

For the luciferase assays, we used HEK-293T cells (ACC 635), which were purchased from the German collection of microorganisms and cell cultures (DSMZ) and authenticated by STR typing by DSMZ. HEK-293T cells were cultured in DMEM (Life Technologies, Darmstadt, Germany) supplemented with 100 U/ml penicillin, 100 µg/ml streptomycin and 10% FCS (Thermo Fisher, Waltham, USA) and subcultured two times a week for less than three months after receipt.

### Transfection of MiRNA and siRNA-mediated transfection

The cells were seeded in 6-well plates (LNCaP Abl EnzR 250,000 cells/well; DuCaP EnzR: 300,000 cells/well) or 96-well plates (LNCaP Abl EnzR 12,000 cells/well; DuCaP EnzR: 15,000 cells/well). The cells were transfected via lipofection (Lipofectamine RNAiMAX; Thermo Fisher Scientific, Darmstadt, Germany) according to the manufacturer’s recommendations at a final concentration of 50 nM of each miRNA or siRNA and incubated for 72 h. The cells were transfected with miRIDIAN microRNA mimics (miRIDIAN micoRNA Human hsa-miR-454-3p, cat. C-301004-03-0005; miRIDIAN microRNA Mimic Negative Control #1, Dharmacon, Horizon Discovery, Cambridge, UK). For MED12 knockdown, ON-TARGETplus SMARTpools were used (ON-TARGET plus nontargeting Control Pool, cat. D-001810-10-05; ON-TARGET plus Human MED12 siRNA, cat. L-009092-00-0005, Dharmacon).

### Cell viability assay

Cell viability was determined in 96-well plates 72 h after transfection via the colorimetric WST-1 assay (Roche Diagnostics, Penzberg, Germany). WST-1 was added (1:10 final dilution) and incubated for 2 h at 37 °C and 5% CO_2_. The absorbance of the samples was measured in a plate reader (440 nm) (Tecan M200Pro, Männedorf, Switzerland) with cell culture medium used as a blank control.

### Western hybridization and densitometry

Protein isolation from cultured cells was performed via RIPA buffer (25 mM Tris-HCl [pH of 8.0], 137 mM NaCl, 10% glycerol, 0.1% SDS, 0.5% sodium deoxycholate, 1% NP40, and 2 mM EDTA [pH of 8.0]) containing protease inhibitors (complete protease inhibitor cocktail, Merck, Darmstadt, Germany). The protein concentrations of the whole-cell protein lysates were measured with a ROTI Quant Universal Kit (Carl Roth GmbH, Karlsruhe, Germany). A total of 15 µg of protein was separated via SDS‒PAGE (8%) and transferred onto a nitrocellulose membrane (GE Healthcare, Frankfurt a.M., Germany) via semidry electroblotting. The membranes were blocked in TBST supplemented with 5% nonfat dry milk (Carl Roth, Germany) for one hour. The membranes were subsequently incubated overnight (with the exception of the GAPDH antibody for 1 h) at 4 °C with the appropriate primary antibodies diluted in 5% TBST blocking solution. The primary antibodies used were monoclonal rabbit MED12 (clone D9K5J; Cell Signaling, Frankfurt a.M., Germany; 1:1000), monoclonal rabbit AR (clone D6F11; Cell Signaling, 1:1000), monoclonal rabbit AR-V7 (clone RM7; RevMAb Biosciences, Hamburg, Germany; 1:1000), monoclonal rabbit c-Myc (clone Y69, Abcam, Cambridge, UK; 1:1000) and monoclonal rabbit GADPH (clone 14C10, Cell Signaling; 1:1000). The secondary anti-rabbit antibody conjugated with horseradish peroxidase was purchased from Jackson ImmunoResearch (Sufolk, UK) and used at a concentration of 1:5000 in 5% TBST blocking solution for one hour at room temperature. An ImageQuant LAS-4000 Imager was used for detection of the chemiluminescence signal. Densitometric analysis of the protein bands was performed via ImageJ software (version 1.52a), with normalization to the level of the housekeeping control protein GAPDH.

### MiRNA expression plasmid and reporter vectors

The sequence inserted for hsa-mir-454 (miRBase accession number: MI0003820), which represents miRNA precursor sequences with additional nucleotides up- and downstream of the precursor sequence, was synthesized by Eurofins Genomics (Ebersberg, Germany) and cloned and inserted into the pSG5 expression plasmid (Agilent Technologies, Santa Clara, California) via EcoRI and BamHI restriction sites. The pSG5-miR-454 expression plasmid harbors the nucleotides 57,215,021–57,215,333[-] of human chromosome 17 (GRCh38/hg38). The cloned sequence of the miR-454 expression plasmid was verified by Sanger sequencing and is shown in Suppl. Table S1. As positive controls for regulatory effects, a sequence insert containing two copies of the complementary sequence for miR-454-3p as well as additional random flanking nucleotides with no self-complementary sites was generated in silico, synthesized by Eurofins Genomics (Ebersberg, Germany) and cloned and inserted into the reporter plasmid pMIR-RNL-TK via the SpeI and SacI restriction sites. The cloned sequence of the miR-454-3p sensor vector was verified by Sanger sequencing and is shown in Suppl. Table S1. The reporter plasmids containing inserts of 443 bp for pMIR-AR_1, 499 bp for pMIR-AR_2, 498 bp for pMIR-AR-V7_1 and 499 bp for pMIR-AR-V7_2 were synthesized, cloned, and inserted into the reporter plasmid pMIR-RNL-TK via the SpeI and SacI restriction sites by GeneArt Gene Synthesis Services (Thermo Fisher, Waltham, USA). The correct cloning of all reporter constructs was verified by Sanger sequencing. A complete list of all 3’UTR sequences, including the respective NM accession numbers, is given in Suppl. Table S1.

### High-throughput MiRNA interaction reporter (HiTmIR) assay

The HiTmIR assay was performed as described previously^61^. In brief, approximately 3.0 × 10^4^ HEK-293T cells were seeded in a 96-well plate via the liquid handling system epMotion 5075 (Eppendorf, Hamburg, Germany). After 24 h, the cells were transfected with 50 ng/well of the reporter plasmid pMIR-RNL-TK, either with or without the 3’UTR insert, or with 200 ng/well of the miR-454 expression plasmid or empty pSG5. After 48 h, the cells were lysed and measured on a GloMax Navigator microplate luminometer (Promega, Madison, WI, USA) with the Dual-Luciferase Reporter Assay System (Promega, Madison, WI, USA). For analysis of the relative light units (RLUs), the firefly luciferase activity of each wild-type 3’UTR reporter plasmid was standardized by the activity of a constitutively expressed renilla luciferase (Firefly/Renilla ratio). The standardized luciferase activity of each wild-type 3’UTR reporter plasmid cotransfected with the miR-454 expression plasmid was normalized to the luciferase activity of the empty reporter vector cotransfected with the miR-454 expression vector. The HiTmIR Assays were performed three times in technical duplicates.

### Northern blot analysis

The overexpression of miR-454-3p and correct processing in HEK-293T cells were verified by Northern blotting. Northern blotting was performed as described previously^25^. Northern blotting was conducted via a radiolabeled DNA probe specific for miR-454-3p (5’TAGTGCAATATTGCTTATAGGGTCCTGTCTC3’). The results of the Northern blot analysis are shown in Suppl. Fig. S1.

### Quantitative real-time PCR

The mRNA transcripts were detected using TaqMan gene expression assays (Thermo Fisher Scientific) according to the manufacturer’s protocol. Briefly, 1 µg of RNA was reverse transcribed using the Maxima cDNA synthesis kit (Thermo Fisher Scientific). The reactions were carried out using the StepOne Plus Real-Time PCR System (Thermo Fisher Scientific) in triplicate in a final volume of 10 µL with cDNA equivalent to 25 ng RNA, using TaqMan gene expression assays (KLK3, Hs02576345_m1; and PCR reagents according to the manufacturer’s instructions. No template controls were included in the reaction plates. Thermal cycling conditions were 95 °C for 20 s, followed by 40 cycles of 95 °C for 1 s and 60 °C for 20 s. GAPDH (Hs99999905_m1, Thermo Fisher Scientific) served as the endogenous reference. Relative mRNA expression levels were calculated according to the dCt method.

### Statistics

The statistical analysis of HiTmIR assays was performed via GraphPad Prism 9 via multiple Welsh’s t test vs. control. The p-values presented are multiplicity adjusted p-values. Changes in protein expression and viability were analyzed by fitting a one-way ANOVA model with post hoc multiple comparisons against the negative control using Dunnett contrasts with adjustment of p values via a single-step adjustment based on a joint T distribution as implemented in the multcomp package for R. For conducting dose‒response analyses, a 4-parameter log-logistic model was fitted, whereby the lower limit was constrained to the maximum detectable effect on viability reduction. The half-effective dose (ED50) was calculated according to the log-logistic model. Model fitting was performed via the drc package. All calculations were conducted with the R statistical framework (ver. 4.2.1; R Foundation for Statistical Computing, Vienna, Austria; https://www.R-project.org/).

## Results

### Luciferase assays revealing AR and AR-V7 as targets of miR-454-3p

**Fig. 1:**
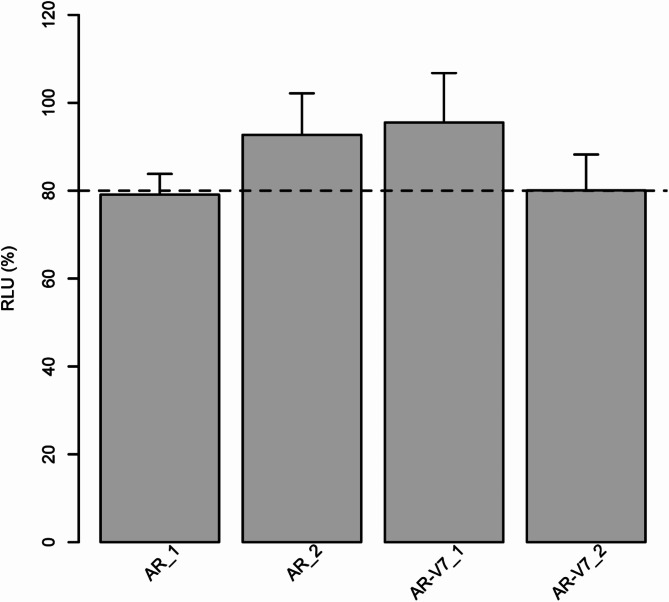
Luciferase assays for identifying putative miR-454-3p binding sites in AR or AR-V7 3’UTRs. The reporter plasmids pMIR-RNL-TK with the androgen receptor 3’UTR AR_1 (AR_1) or with the androgen receptor splice variant 7 3’UTR AR-V7_V2 (AR-V7_2) showed an approximately 20% reduction in luciferase activity after cotransfection with a pSG5-miR-454-3p expression plasmid in HEK-293T cells. AR and AR-V7 are identified as target genes for miR-454-3p. All values represent the relative luciferase activity normalized to that of the corresponding negative control. The dashed line indicates the multiple test-corrected threshold for a significant reduction in luciferase activity. All the experiments represent three independent biological replicates.

Two luciferase reporter plasmids with androgen receptor 3’UTRs (AR_1 or AR_2) and two with androgen receptor splice variant 7 3’UTRs (AR-V7_1 or AR-V7_2) were cotransfected with the miR-454-3p expression plasmid in HEK-293T cells. In the presence of miR-454-3p, the 3’UTR constructs AR_1 and AR-V7_2 were both able to significantly suppress the activity of the reporter gene by approximately 20% (Fig. 1). However, the 3’UTR constructs AR-2 and AR-V7_1 did not affect reporter gene activity (Fig. 1). Taken together, we confirmed that the miR-454-3p binding sites AR_1 and AR-V7_2 are regulatory targets of this miRNA.

### Resistance profile of the enzalutamide-resistant cell lines

To record the resistance profiles of LNCaP Abl EnzR and DuCaP EnzR cells, the cells were incubated with serial dilutions of enzalutamide, covering the concentration range of 4–128 µM. After 72 h, the cell viability was determined via the colorimetric WST assay. After log-logistic regression models were fitted to the data points, we determined the half-effective dose (ED50) for enzalutamide. The LNCaP Abl EnzR cell line presented an ED50 of 32.4 µM (95% CI: 28.5–36.2 µM) and was more sensitive than the DuCaP EnzR cell line, with an ED50 of 60.8 µM (95% CI: 52.1–69.6 µM; Suppl. Fig. S2). For further analysis of cell viability, we selected three distinct conditions: (i) without enzalutamide, (ii) cell line-specific maintenance concentrations and (iii) cell line-specific ED50 concentrations.

### Effects of siMED12, miR-454-3p, or their combination on the protein expression of MED12, AR, AR-V7 and c-Myc

To gain more insight into the tumor-relevant pathways affected by MED12 knockdown or miRNA modulation, we determined the protein expression of MED12, the androgen receptor (AR), the androgen receptor splice variant V7 (AR-V7) and c-Myc.

**Fig. 2:**
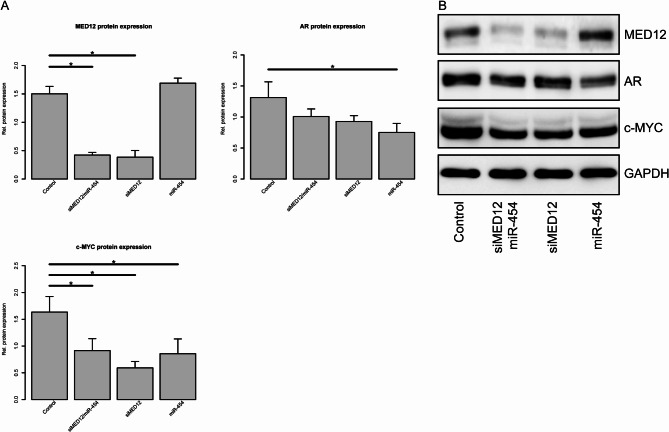
Effects of siMED12 and miR-454 on protein expression in LNCaP Abl EnzR cells. Western hybridization for protein detection of MED12, AR, and c-Myc in LNCaP Abl EnzR cells transfected with siMED12, miR-454-3p or combined siMED12/miR-454-3p. (**A**) Mean protein expression of three independent experiments. All the experiments represent three independent biological replicates. (**B**) Representative Western hybridization results.

**Fig. 3:**
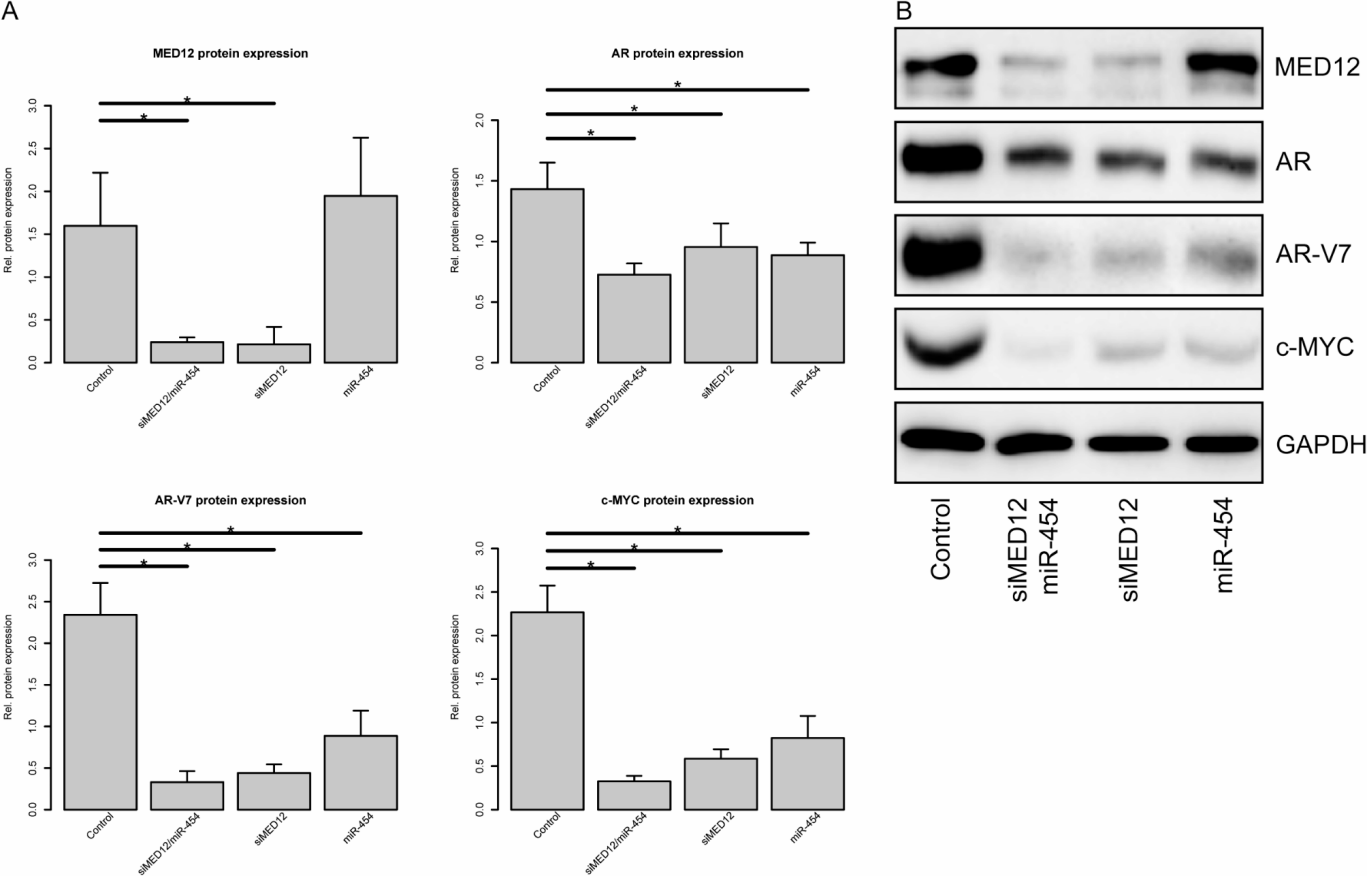
Effects of siMED12 and miR-454 on protein expression in DuCaP EnzR cells. Western hybridization for protein detection of MED12, AR, AR-V7 and c-Myc in DuCaP EnzR cells transfected with siMED12, miR-454-3p or combined siMED12/miR-454-3p. (**A**) Mean protein expression of three independent experiments. All the experiments represent three independent biological replicates. (**B**) Representative Western hybridization results.

MED12 protein expression was effectively and significantly reduced after MED12 knockdown by siMED12 in both cell lines (Figs. 2 and 3). The miRNA miR-454 was not able to reduce MED12 protein under any conditions (Figs. 2 and 3). Compared with the single siMED12 treatment, the combination of siMED12 and miR-454 significantly reduced MED12 protein expression in both cell lines. No additive or synergistic effects of the combination treatment were detected (Figs. 2 and 3). Taken together, these findings indicate that the siRNA-mediated knockdown of MED12 efficiently reduced MED12 protein expression, as expected. The combination of siMED12 with miR-454 had no additive or synergistic effects.

AR protein expression was reduced after MED12 knockdown in both cell lines (Figs. 2 and 3). Additionally, a significant reduction in AR protein expression was observed after miR-454 treatment in both cell lines (Figs. 2 and 3). The combination of siMED12 and miR-454 significantly reduced the AR protein level only in the DuCaP EnzR cell line (Fig. 3) but not in the LNCaP Abl EnzR cell line (Fig. 2). Taken together, these findings indicate that ectopic miR-454 could significantly reduce AR protein expression in both cell lines. The effect of the combination treatment (siMED12/miR-454) on AR protein expression was restricted to the DuCaP EnzR cell line. Interestingly, the mRNA expression of the AR target gene KLK3 was significantly reduced after miR-454, siMED12 and mir-454/siMED12 treatment in the LNCaP Abl EnzR cell line but not in the DuCaP EnzR cell line (Suppl. Fig. S3) suggesting a different molecular behavior of AR-dependent (LNCaP Abl EnzR) and AR-independent (DuCaP EnzR) cell lines.

AR-V7 protein is expressed only in the DuCaP EnzR cell line but not in the LNCaP Abl EnzR cell line. AR-V7 protein expression was significantly reduced by all treatment modalities, including siMED12, miR-454 and the combination of siMED12/miR-454, in the DuCaP EnzR cell line (Fig. 3). Taken together, AR-V7 protein expression, which is only present in the DuCaP EnzR cell line, was reduced after miR-454 treatment, and the combination of siMED12/miR-454 followed the pattern observed for the AR protein.

The c-Myc protein level was significantly reduced by MED12 knockdown, miR-454 or combined siMED12/mir-454 treatment in the DuCaP EnzR cell line (Fig. 3) and less pronounced but still significant in the LNCaP Abl EnzR cell line (Fig. 2). Interestingly, the combination of siMED12 and miR-454 had an additive effect on c-Myc protein reduction in the DuCaP EnzR cell line (Fig. 3) but not in the LNCaP Abl EnzR cell line (Fig. 2). Overall, the c-Myc protein level was reduced after siMED12, miR-454 and the combination of siMED12/mir-454 in both EnzR cell lines. A reduction in c-Myc could be of future therapeutic importance since c-Myc is considered a critical cancer stem cell transcription factor that strongly affects therapy response and resistance^55^.

To summarize the effects on protein expression, the siRNA-mediated knockdown of MED12 was most effective and significantly reduced MED12, AR, AR-V7 and c-Myc protein levels in the DuCaP EnzR cell line. Similarly, siMED12 was also effective and significantly reduced MED12 and c-Myc but did not significantly reduce AR protein levels in the LNCaP Abl EnzR cell line. In addition, single treatment with miR-454 reduced AR and c-Myc protein expression in both EnzR cell lines and AR-V7 expression in the DuCaP EnzR cell line. The effects of the combination treatments on protein expression can be attributed to the siMED12 component.

### Effects of miR-454-3p, siMED12 and their combination on cell viability

To test the effects of MED12 knockdown or miR-454 modulation on cell line viability, we transfected the LNCaP Abl EnzR and DuCaP EnzR cell lines with siMED12, miR-454, or a combination of siMED12/miR-454. Moreover, we sought to determine the importance of the continued selective pressure by using enzalutamide with the maintenance enzalutamide concentrations (LNCaP Abl EnzR: 13 µM and DuCaP EnzR: 8 µM) or removing the selective pressure.

**Fig. 4:**
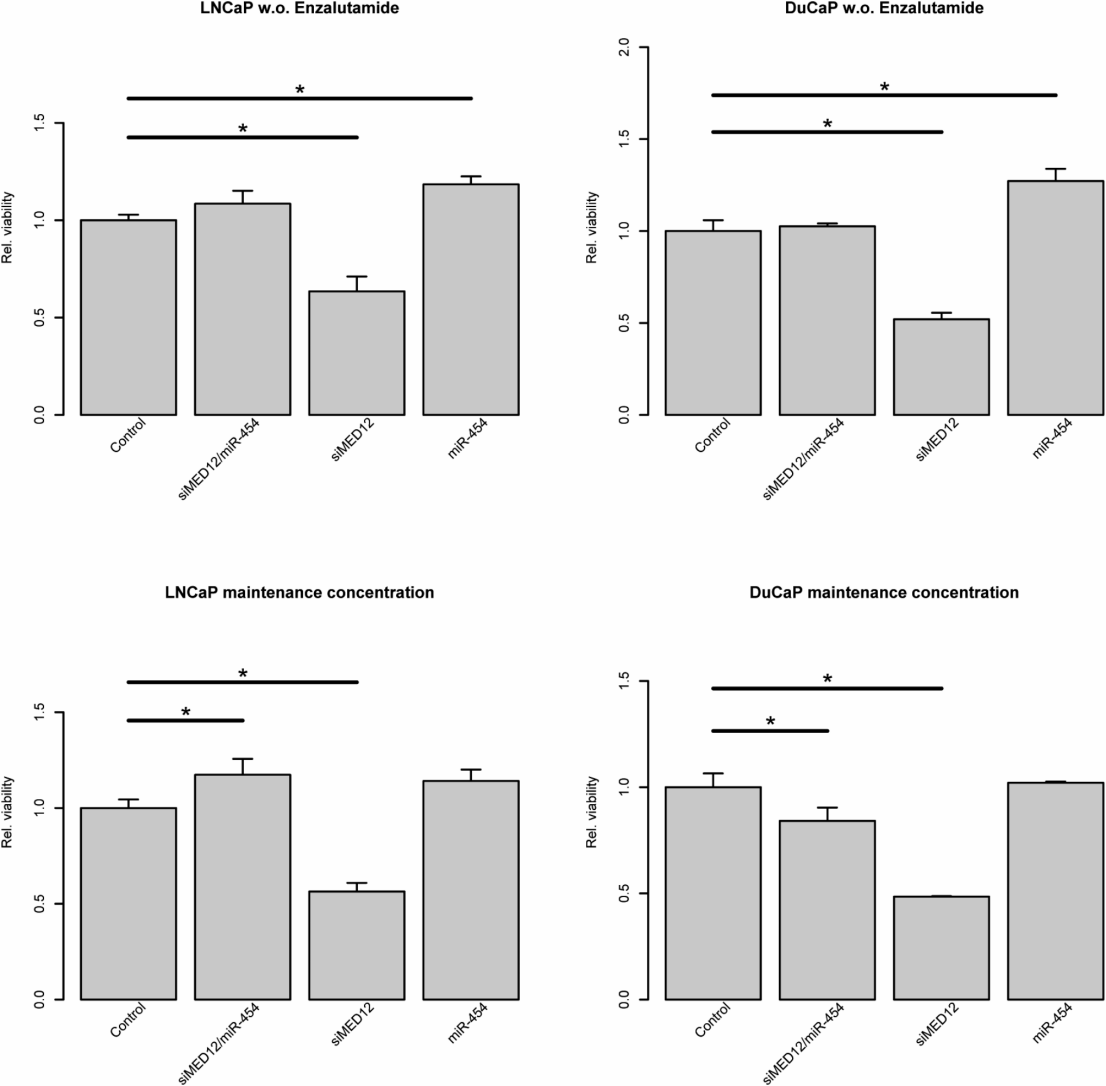
Impact of siMED12 and miR-454 on cell viability. Relative viability of LNCaP Abl EnzR and DuCaP EnzR cells cultured without (w.o.) enzalutamide (upper row) or under maintenance concentrations of enzalutamide (lower row) and transfected with siMED12, miR-454-3p or combined siMED12/miR-454-3p. All the experiments represent three independent biological replicates.

The knockdown of MED12 caused a significant reduction in the viability of the LNCaP Abl EnzR and DuCaP EnzR cell lines, irrespective of the enzalutamide conditions (Fig. 4). In contrast, miRNA overexpression by transfection of synthetic miR-454-3p did not result in a reduction in cell viability and even led to a significant increase in cell viability in the LNCaP Abl EnzR and DuCaP EnzR cell lines, particularly under conditions where the selective pressure was removed.

To test for an additive or even synergistic effect of MED12 knockdown and miRNA modulation, we next used a combination of siMED12 and miR-454-3p. The combination of siMED12/miR-454 resulted in a significant increase in cell viability in the LNCaP Abl EnzR but a significant decrease in cell viability in the DuCaP EnzR cell lines. Notably, the combination of siMED12/miR-454 resulted in this effect under the condition of enzalutamide maintenance concentration (Fig. 4).

We tested the effect on cell viability and challenged the cells with individual ED50 doses of enzalutamide, the LNCaP Abl EnzR cell line (ED50: 32.4 µM) and the DuCaP EnzR cell line (ED50: 60.8 µM). As before, in both cell lines, cell viability was significantly lower after MED12 knockdown than that in the control when challenged with ED50 doses of enzalutamide (Suppl. Fig. S4). Interestingly, the reduction in cell viability caused by MED12 knockdown was more pronounced when the cells were challenged with ED50 doses of enzalutamide than with maintenance concentrations or without enzalutamide.

Overall, we detected a strongly significant reduction in cell viability after transfection with siMED12 in the LNCaP Abl EnzR and DuCaP EnzR cell lines at all three enzalutamide concentrations, i.e., without enzalutamide and at the maintenance concentration ED50 doses.

## Discussion

The Mediator complex component MED12 and the microRNA miR-454-3p have been described as diagnostic and/or prognostic factors for PCa patients^21,50–53^. However, their functional effects on cell viability, interactions and effects on the protein expression of MED12, AR, AR-V7 and c-Myc in the context of enzalutamide resistance have not been comprehensively studied. We selected two enzalutamide-resistant cell lines, LNCaP Abl EnzR and DuCaP EnzR, for our study because they recapitulate real-life clinical situations involving enzalutamide resistance and are suggested as preclinical models for castration-resistant PCa (CRPC) patients^58,63^.

We found for the first time in luciferase assays that miR-454-3p targets AR and the AR splice variant 7 (AR-V7) but not MED12. Our findings help elucidate the role of miR-454-3p in PCa. To date, only the tumor suppressor gene NDRG (N-myc downstream-regulated gene 2) has been described as a target of miR-454-3p in PCa, and via NDRG repression, miR-454-3p is able to regulate the protein expression of β-catenin and the activation of WNT signaling^51^.

To gain a better understanding of the regulatory effects on several tumor-related signaling pathways, we studied the protein expression of MED12, AR, AR-V7 and c-Myc via Western hybridization. The knockdown of MED12 had the most effective and significant effect on reducing MED12, AR, AR-V7 and c-Myc protein expression in the DuCaP EnzR cell line and had a lesser and only partially significant effect on MED12, AR and c-Myc expression in the LNCaP Abl EnzR cell line. However, siMED12 did not substantially decrease androgen-stimulated probasin-luciferase reporter activity in LNCaP cells^64^. We recently showed that siMED12 could inhibit c-Myc and AR-V7 protein expression in 22Rv1 PCa cells, this reduction in AR signaling was accompanied by a reduced expression of specific AR-target genes such as KLK3, FKBP5, TMPRSS2^23^. The depletion of MED12 represses c-myc-associated genes in colon cancer cells^65^, and the inhibition of MED12 reduces c-Myc expression in colon cancer^66^. Gonzalez et al. reported that a reduction in MED12 inhibits EGFR signaling and can upregulate the expression of P21/WAF1^22^, which is well known to play a role in PCa biology^67^. These findings and our results suggest that the inhibition of MED12 is a therapeutic option, at least in PCa. However, reports indicate that the suppression or reduction of MED12 protein expression may be related to different resistance mechanisms against chemotherapy, MEK/BRAF inhibitors or tyrosine kinase inhibitors^22,65^. Overall, further studies are necessary to analyze and evaluate the therapeutic potential of MED12 inhibition in PCa.

In addition, the transfection of synthetic miR-454 reduced AR and c-Myc protein expression in both EnzR cell lines and AR-V7 expression in the DuCaP EnzR cell line. Several reports describe a mutual interaction between AR and c-Myc. Grad et al. identified a myc consensus site in the AR coding region that is involved in AR binding and androgen-mediated upregulation of AR mRNA^68^. This finding was further supported by Zhou et al., who reported a c-Myc binding site within the AR gene. They reported that by altering histone modifications at the c-Myc binding site within the AR gene via RNF8 (RING finger E3 ligase 8), c-Myc-induced AR transcription can be upregulated^69^. On the other hand, Lamb et al. reported that persistent AR activity in advanced PCa regulates cell cycle activity, steroid biosynthesis and anabolic metabolism in conjunction with regulatory cofactors, such as the E2F family, signal transducer and activator of transcription (STAT) and c-Myc transcription factors^70^. Overall, we report that siMED12 and miR-454 are able to inhibit AR, AR-V7 (only in DuCaP EnzR cells) and c-Myc protein expression in DuCaP EnzR and LNCaP Abl EnzR cells. However, we were unable to detect additive effects after combined treatment with siMED12/miR-454. These findings suggest that both proteins functionally affect the AR/c-Myc pathway. Furthermore, the reduction in the level of the oncogenic c-Myc protein after miR-454-3p treatment highlights the function of miR-454-3p. However, these findings indicate that miR-454-3p can, depending on the target gene, function as an oncogene or tumor suppressor in PCa.

We analyzed the effects of MED12 (siMED12) knockdown, miR-454 overexpression, and the combination of siMED12/miR-454 on cell viability. The overexpression of miR-454 resulted in significantly increased viability of LNCaP Abl EnzR and DuCaP EnzR cells. Previously, Fu et al. reported that the overexpression of miR-454 markedly promoted the proliferation and invasion of androgen-independent PCa cell lines^51^. This finding is in accordance with the previously suggested role of miR-454-3p as an oncogene in PCa^50–53^.

In contrast, MED12 knockdown resulted in a significant reduction in cell viability in both cell lines. This effect was observed irrespective of the concentration of enzalutamide in the cultivation media. Adler et al. previously reported that MED12 inhibition reduced cell proliferation in androgen-dependent and androgen-independent PCa cell lines and their 3D spheroids^21,23^, which is in accordance with the findings of this study. Overall, the reduction in cell viability caused by siMED12 was shown for the first time in this study in EnzR PCa cell lines.

Most interestingly, knockdown of MED12 by siRNA significantly reduced the viability of both EnzR cell lines. In addition, this treatment significantly decreased the protein expression of AR and c-Myc in DuCaP EnzR cells but not significantly in LNCaP Abl EnzR cells. Furthermore, siMED12 significantly reduced AR-V7 protein expression, which is considered a resistance mechanism against androgen deprivation therapy^71^, in DuCaP EnzR cells. Our findings suggest that MED12 is a potential target for future PCa treatment in conjunction with enzalutamide resistance.

Interestingly, the results of miR-454 overexpression revealed a decrease in AR protein expression (miR-454 and the siMED12/miR-454 combination) in both EnzR cell lines. Knocking down AR expression by siRNA can induce PI3K-independent activation of Akt, an important positive regulator of cell proliferation and survival^72^. In our study, the suppression of the AR protein by miR-454-3p or the combination of siMED12/miR-454 was accompanied by an increase in the viability of the EnzR cells. These findings may be relevant for PCa treatment since bipolar androgen treatment (BAT) of PCa patients with rapid cycling between high and low serum testosterone, i.e., androgen deprivation and androgen supply, has been shown to be successful in PCa treatment^73^. In addition, PCa patients treated with BAT can be rechallenged with enzalutamide^73^.

In summary, we identified AR and AR-V7 as direct targets of miR-454-3p. With the downregulation of AR, AR-V7 and c-Myc protein expression by miR-454-3p, we could add knowledge to the molecular mechanisms involved in PCa biology. Our study of EnzR PCa cell lines suggests that MED12 is a potential target for future PCa treatment despite enzalutamide resistance.

## Electronic supplementary material

Below is the link to the electronic supplementary material.

Supplementary Material 1

Supplementary Material 2

## Data Availability

Data is provided within the manuscript or supplementary information files. The detailed datasets used and analyzed during the present study are available from the corresponding author upon reasonable request.

## References

[1] Bray, F. et al. Global cancer statistics 2022: GLOBOCAN estimates of incidence and mortality worldwide for 36 cancers in 185 countries. *CA Cancer J. Clin.* **74**, 229–263 (2024). 38572751 10.3322/caac.21834

[2] Visakorpi, T. et al. In vivo amplification of the androgen receptor gene and progression of human prostate cancer. *Nat. Genet.* **9**, 401–406 (1995). 7795646 10.1038/ng0495-401

[3] Santer, F. R., Erb, H. H. & McNeill, R. V. Therapy escape mechanisms in the malignant prostate. *Semin Cancer Biol.* **35**, 133–144 (2015). 26299608 10.1016/j.semcancer.2015.08.005

[4] Hörnberg, E. et al. Expression of androgen receptor splice variants in prostate cancer bone metastases is associated with castration-resistance and short survival. *PLoS ONE*. **6**, e19059 (2011). 21552559 10.1371/journal.pone.0019059 PMC3084247

[5] Kallio, H. M. L. et al. Constitutively active androgen receptor splice variants AR-V3, AR-V7 and AR-V9 are co-expressed in castration-resistant prostate cancer metastases. *Br. J. Cancer*. **119**, 347–356 (2018). 29988112 10.1038/s41416-018-0172-0 PMC6070921

[6] Antonarakis, E. S. et al. Androgen receptor splice variant 7 and efficacy of taxane chemotherapy in patients with metastatic Castration-Resistant prostate cancer. *JAMA Oncol.* **1**, 582–591 (2015). 26181238 10.1001/jamaoncol.2015.1341 PMC4537351

[7] Antonarakis, E. S. et al. Clinical significance of androgen receptor splice Variant-7 mRNA detection in Circulating tumor cells of men with metastatic Castration-Resistant prostate cancer treated with First- and Second-Line abiraterone and enzalutamide. *J. Clin. Oncol.* **35**, 2149–2156 (2017). 28384066 10.1200/JCO.2016.70.1961 PMC5493048

[8] Kornberg, R. D. Mediator and the mechanism of transcriptional activation. *Trends Biochem. Sci.* **30**, 235–239 (2005). 15896740 10.1016/j.tibs.2005.03.011

[9] Ding, N. et al. Mediator links epigenetic Silencing of neuronal gene expression with x-linked mental retardation. *Mol. Cell.* **31**, 347–359 (2008). 18691967 10.1016/j.molcel.2008.05.023 PMC2583939

[10] Taatjes, D. J. The human mediator complex: A versatile, genome-wide regulator of transcription. *Trends Biochem. Sci.* **35**, 315–322 (2010). 20299225 10.1016/j.tibs.2010.02.004 PMC2891401

[11] Allen, B. L. & Taatjes, D. J. The mediator complex: A central integrator of transcription. *Nat. Rev. Mol. Cell. Biol.* **16**, 155–166 (2015). 25693131 10.1038/nrm3951 PMC4963239

[12] Bourbon, H. M. Comparative genomics supports a deep evolutionary origin for the large, four-module transcriptional mediator complex. *Nucleic Acids Res.* **36**, 3993–4008 (2008). 18515835 10.1093/nar/gkn349 PMC2475620

[13] Malik, S. & Roeder, R. G. The metazoan mediator co-activator complex as an integrative hub for transcriptional regulation. *Nat. Rev. Genet.* **11**, 761–772 (2010). 20940737 10.1038/nrg2901 PMC3217725

[14] Weber, H. & Garabedian, M. J. The mediator complex in genomic and non-genomic signaling in cancer. *Steroids* **133**, 8–14 (2018). 29157917 10.1016/j.steroids.2017.11.007 PMC5864542

[15] Jin, F., Claessens, F. & Fondell, J. D. Regulation of androgen receptor-dependent transcription by coactivator MED1 is mediated through a newly discovered noncanonical binding motif. *J. Biol. Chem.* **287**, 858–870 (2012). 22102282 10.1074/jbc.M111.304519 PMC3256875

[16] Liu, G. et al. MED1 mediates androgen receptor splice variant induced gene expression in the absence of ligand. *Oncotarget* **6**, 288–304 (2015). 25481872 10.18632/oncotarget.2672 PMC4381595

[17] Vijayvargia, R., May, M. S. & Fondell, J. D. A coregulatory role for the mediator complex in prostate cancer cell proliferation and gene expression. *Cancer Res.* **67**, 4034–4041 (2007). 17483314 10.1158/0008-5472.CAN-06-3039

[18] Jin, F. et al. ERK and AKT signaling drive MED1 overexpression in prostate cancer in association with elevated proliferation and tumorigenicity. *Mol. Cancer Res.* **11**, 736–747 (2013). 23538858 10.1158/1541-7786.MCR-12-0618 PMC5838364

[19] Barbieri, C. E. et al. Exome sequencing identifies recurrent SPOP, FOXA1 and MED12 mutations in prostate cancer. *Nat. Genet.* **44**, 685–689 (2012). 22610119 10.1038/ng.2279 PMC3673022

[20] Stoehr, R. et al. Lack of evidence for frequent MED12 p.L1224F mutation in prostate tumours from Caucasian patients. *J. Pathol.* **230**, 453–456 (2013). 23661306 10.1002/path.4208

[21] Adler, D. et al. MED12 overexpression is a frequent event in castration-resistant prostate cancer. *Endocr. Relat. Cancer*. **21**, 663–675 (2014). 24938407 10.1530/ERC-14-0171

[22] Gonzalez, C. G., Akula, S. & Burleson, M. The role of mediator subunit 12 in tumorigenesis and cancer therapeutics. *Oncol. Lett.* **23**, 74 (2022). 35111243 10.3892/ol.2022.13194 PMC8771631

[23] Andolfi, C. et al. MED12 and CDK8/19 modulate androgen receptor activity and enzalutamide response in prostate cancer. *Endocrinology* **165**. (2024). 10.1210/endocr/bqae114 PMC11398899 39253786

[24] Esquela-Kerscher, A. & Slack, F. J. Oncomirs—microRNAs with a role in cancer. *Nat. Rev. Cancer*. **6**, 259–269 (2006). 16557279 10.1038/nrc1840

[25] Slack, F. J. & Weidhaas, J. B. MicroRNAs as a potential magic bullet in cancer. *Future Oncol.* **2**, 73–82 (2006). 16556074 10.2217/14796694.2.1.73

[26] Fabbri, M., Ivan, M., Cimmino, A., Negrini, M. & Calin, G. A. Regulatory mechanisms of MicroRNAs involvement in cancer. *Expert Opin. Biol. Ther.* **7**, 1009–1019 (2007). 17665990 10.1517/14712598.7.7.1009

[27] Fabris, L. et al. The potential of MicroRNAs as prostate cancer biomarkers. *Eur. Urol.* **70**, 312–322 (2016). 26806656 10.1016/j.eururo.2015.12.054 PMC4927364

[28] Catto, J. W. et al. MicroRNA in prostate, bladder, and kidney cancer: A systematic review. *Eur. Urol.* **59**, 671–681 (2011). 21296484 10.1016/j.eururo.2011.01.044

[29] Fendler, A., Stephan, C., Yousef, G. M. & Jung, K. MicroRNAs as regulators of signal transduction in urological tumors. *Clin. Chem.* **57**, 954–968 (2011). 21632885 10.1373/clinchem.2010.157727

[30] Puhr, M. et al. Epithelial-to-mesenchymal transition leads to docetaxel resistance in prostate cancer and is mediated by reduced expression of miR-200c and miR-205. *Am. J. Pathol.* **181**, 2188–2201 (2012). 23041061 10.1016/j.ajpath.2012.08.011

[31] Wach, S. et al. MicroRNA profiles of prostate carcinoma detected by multiplatform MicroRNA screening. *Int. J. Cancer*. **130**, 611–621 (2012). 21400514 10.1002/ijc.26064

[32] Hart, M. et al. Comparative MicroRNA profiling of prostate carcinomas with increasing tumor stage by deep sequencing. *Mol. Cancer Res.* **12**, 250–263 (2014). 24337069 10.1158/1541-7786.MCR-13-0230

[33] Gujrati, H., Ha, S. & Wang, B. D. Deregulated MicroRNAs involved in prostate cancer aggressiveness and treatment resistance mechanisms. *Cancers (Basel)* **15**. (2023). 10.3390/cancers15123140 PMC10296615 37370750

[34] Khan, M. M., Sharma, V. & Serajuddin, M. Emerging role of MiRNA in prostate cancer: A future era of diagnostic and therapeutics. *Gene* **888**, 147761 (2023). 37666374 10.1016/j.gene.2023.147761

[35] Tavares, I., Morais, M., Dias, F., Medeiros, R. & Teixeira, A. L. Deregulated MiRNAs in enzalutamide resistant prostate cancer: A comprehensive review of key molecular alterations and clinical outcomes. *Biochim. Biophys. Acta Rev. Cancer*. **1879**, 189067 (2024). 38160898 10.1016/j.bbcan.2023.189067

[36] Wang, S. et al. MiR-454-3p and miR-374b-5p suppress migration and invasion of bladder cancer cells through targetting ZEB2. *Biosci. Rep.* **38**. (2018). 10.1042/BSR20181436 PMC6435551 30352837

[37] An, Y. et al. miR-454 suppresses the proliferation and invasion of ovarian cancer by targeting E2F6. *Cancer Cell. Int.* **20**, 237 (2020). 32536825 10.1186/s12935-020-01300-0 PMC7291497

[38] Liao, H. et al. miR–454–3p inhibits non–small cell lung cancer cell proliferation and metastasis by targeting TGFB2. *Oncol. Rep.* **45**. (2021). 10.3892/or.2021.8018 PMC8020204 33760169

[39] Zhang, Q., Wei, J., Li, N. & Liu, B. LINC00839 promotes neuroblastoma progression by sponging miR-454-3p to Up-Regulate NEUROD1. *Neurochem. Res.* **47**, 2278–2293 (2022). 35606572 10.1007/s11064-022-03613-0

[40] Wang, X. et al. MiR-454-3p promotes apoptosis and autophagy of AML cells by targeting ZEB2 and regulating AKT/mTOR pathway. *Hematology* **28**, 2223874 (2023). 37313984 10.1080/16078454.2023.2223874

[41] de Melo Maia, B. et al. MicroRNA portraits in human vulvar carcinoma. *Cancer Prev. Res. (Phila)*. **6**, 1231–1241 (2013). 24048714 10.1158/1940-6207.CAPR-13-0121

[42] Yu, L. et al. miR-454 functions as an oncogene by inhibiting CHD5 in hepatocellular carcinoma. *Oncotarget* **6**, 39225–39234 (2015). 26287602 10.18632/oncotarget.4407 PMC4770768

[43] Li, Q., Liu, J., Meng, X., Pang, R. & Li, J. MicroRNA-454 May function as an oncogene via targeting AKT in triple negative breast cancer. *J. Biol. Res. (Thessalon)*. **24**, 10 (2017). 28795052 10.1186/s40709-017-0067-x PMC5541748

[44] Lu, L. et al. MicroRNAs in the prognosis of triple-negative breast cancer: A systematic review and meta-analysis. *Med. (Baltim).* **96**, e7085 (2017). 10.1097/MD.0000000000007085 PMC5459744 28562579

[45] Ren, L. et al. MiR-454-3p-Mediated Wnt/beta-catenin signaling antagonists suppression promotes breast cancer metastasis. *Theranostics* **9**, 449–465 (2019). 30809286 10.7150/thno.29055 PMC6376193

[46] Liu, H. et al. Silencing miR-454 suppresses cell proliferation, migration and invasion via directly targeting MECP2 in renal cell carcinoma. *Am. J. Transl. Res.* **12**, 4277–4289 (2020). 32913504 PMC7476129

[47] Shen, G. et al. miR-454-3p promotes of human glioma cell growth by targeting EGR3. *Exp. Ther. Med.* **18**, 4031–4039 (2019). 31641382 10.3892/etm.2019.8048 PMC6796382

[48] Shao, N. et al. miR-454-3p is an Exosomal biomarker and functions as a tumor suppressor in glioma. *Mol. Cancer Ther.* **18**, 459–469 (2019). 30413650 10.1158/1535-7163.MCT-18-0725

[49] Daniel, R. et al. A panel of MicroRNAs as diagnostic biomarkers for the identification of prostate cancer. *Int. J. Mol. Sci.* **18**. (2017). 10.3390/ijms18061281 PMC5486103 28621736

[50] Nam, R. K. et al. Identification and validation of a five MicroRNA signature predictive of prostate cancer recurrence and metastasis: A cohort study. *J. Cancer*. **6**, 1160–1171 (2015). 26516365 10.7150/jca.13397 PMC4615353

[51] Fu, Q. et al. Suppression of microRNA-454 impedes the proliferation and invasion of prostate cancer cells by promoting N-myc downstream-regulated gene 2 and inhibiting WNT/beta-catenin signaling. *Biomed. Pharmacother*. **97**, 120–127 (2018). 29080452 10.1016/j.biopha.2017.10.115

[52] Oh-Hohenhorst, S. J. & Lange, T. Role of metastasis-related MicroRNAs in prostate cancer progression and treatment. *Cancers (Basel)* **13** (2021). 10.3390/cancers13174492 PMC8431208 34503302

[53] Bozgeyik, E. & Ceylan, O. Distinct expression signatures of miR-130a, miR-301a, miR-454 in formalin fixed paraffin embedded tissue samples of prostate cancer patients. *Pathol. Res. Pract.* **234**, 153897 (2022). 35468339 10.1016/j.prp.2022.153897

[54] Horoszewicz, J. S. et al. The LNCaP cell line–a new model for studies on human prostatic carcinoma. *Prog. Clin. Biol. Res.* **37**, 115–132 (1980). 7384082

[55] Horoszewicz, J. S. et al. LNCaP model of human prostatic carcinoma. *Cancer Res.* **43**, 1809–1818 (1983). 6831420

[56] Veldscholte, J. et al. A mutation in the ligand binding domain of the androgen receptor of human LNCaP cells affects steroid binding characteristics and response to anti-androgens. *Biochem. Biophys. Res. Commun.* **173**, 534–540 (1990). 2260966 10.1016/s0006-291x(05)80067-1

[57] Culig, Z. et al. Switch from antagonist to agonist of the androgen receptor bicalutamide is associated with prostate tumour progression in a new model system. *Br. J. Cancer*. **81**, 242–251 (1999). 10496349 10.1038/sj.bjc.6690684 PMC2362859

[58] Hoefer, J. et al. Critical role of androgen receptor level in prostate cancer cell resistance to new generation antiandrogen enzalutamide. *Oncotarget* **7**, 59781–59794 (2016). 27486973 10.18632/oncotarget.10926 PMC5312348

[59] Hu, R. et al. Ligand-independent androgen receptor variants derived from splicing of cryptic exons signify hormone-refractory prostate cancer. *Cancer Res.* **69**, 16–22 (2009). 19117982 10.1158/0008-5472.CAN-08-2764 PMC2614301

[60] Lee, Y. G. et al. Establishment and characterization of a new human prostatic cancer cell line: DuCaP. *Vivo* **15**, 157–162 (2001). 11317521

[61] Hart, M. et al. Experimental capture of MiRNA targetomes: Disease-specific 3’UTR library-based MiRNA targetomics for Parkinson’s disease. *Exp. Mol. Med.* **56**, 935–945 (2024). 38556547 10.1038/s12276-024-01202-5 PMC11059366

[62] Lee, W. K. & Cheng, S. Y. Targeting transcriptional regulators for treatment of anaplastic thyroid cancer. *J. Cancer Metastasis Treat.* **7** (2021). 10.20517/2394-4722.2021.58 PMC8577520 34761120

[63] Moll, J. M. et al. Cell line characteristics predict subsequent resistance to androgen Receptor-Targeted agents (ARTA) in preclinical models of prostate cancer. *Front. Oncol.* **12**, 877613 (2022). 35769712 10.3389/fonc.2022.877613 PMC9234122

[64] Imberg-Kazdan, K. et al. A genome-wide RNA interference screen identifies new regulators of androgen receptor function in prostate cancer cells. *Genome Res.* **23**, 581–591 (2013). 23403032 10.1101/gr.144774.112 PMC3613576

[65] Huang, S. et al. MED12 controls the response to multiple cancer drugs through regulation of TGF-beta receptor signaling. *Cell* **151**, 937–950 (2012). 23178117 10.1016/j.cell.2012.10.035 PMC3672971

[66] Kuuluvainen, E., Domenech-Moreno, E., Niemela, E. H. & Makela, T. P. Depletion of mediator kinase module subunits represses Superenhancer-Associated genes in colon cancer cells. *Mol. Cell. Biol.* **38**. (2018). 10.1128/MCB.00573-17 PMC5954191 29507187

[67] Agarwal, R. Cell signaling and regulators of cell cycle as molecular targets for prostate cancer prevention by dietary agents. *Biochem. Pharmacol.* **60**, 1051–1059 (2000). 11007941 10.1016/s0006-2952(00)00385-3

[68] Grad, J. M., Dai, J. L., Wu, S. & Burnstein, K. L. Multiple androgen response elements and a Myc consensus site in the androgen receptor (AR) coding region are involved in androgen-mediated up-regulation of AR messenger RNA. *Mol. Endocrinol.* **13**, 1896–1911 (1999). 10551783 10.1210/mend.13.11.0369

[69] Zhou, T. et al. RNF8 up-regulates AR/ARV7 action to contribute to advanced prostate cancer progression. *Cell. Death Dis.* **13**, 352 (2022). 35428760 10.1038/s41419-022-04787-9 PMC9012884

[70] Lamb, A. D., Massie, C. E. & Neal, D. E. The transcriptional programme of the androgen receptor (AR) in prostate cancer. *BJU Int.* **113**, 358–366 (2014). 24053777 10.1111/bju.12415

[71] Luna Velez, M. V., Verhaegh, G. W., Smit, F., Sedelaar, J. P. M. & Schalken, J. A. Suppression of prostate tumor cell survival by antisense oligonucleotide-mediated Inhibition of AR-V7 mRNA synthesis. *Oncogene* **38**, 3696–3709 (2019). 30664691 10.1038/s41388-019-0696-7 PMC6756119

[72] Cohen, M. B. & Rokhlin, O. W. Mechanisms of prostate cancer cell survival after Inhibition of AR expression. *J. Cell. Biochem.* **106**, 363–371 (2009). 19115258 10.1002/jcb.22022

[73] Denmeade, S. R. et al. TRANSFORMER: A randomized phase II study comparing bipolar androgen therapy versus enzalutamide in asymptomatic men with Castration-Resistant metastatic prostate cancer. *J. Clin. Oncol.* **39**, 1371–1382 (2021). 33617303 10.1200/JCO.20.02759 PMC8274807

