# Supplementary material for: The MicroRNA miR-454 and the mediator complex component MED12 are regulators of the androgen receptor pathway in prostate cancer
Source: Sci Rep. 2025 Mar 25;15:10272. doi: 10.1038/s41598-025-95250-0 (PMC11937531; doi:10.1038/s41598-025-95250-0)
Supplement: Supplementary file 2 — Supplementary Material 2 [file 41598_2025_95250_MOESM2_ESM.docx]

Supplementary Figures

The microRNA miR-454 and the Mediator Complex component MED12 are regulators of the androgen receptor pathway in prostate cancer

Juan Guzman ^1,2^, Martin Hart ^3^, Katrin Weigelt ^1,2^, Angela Neumann ^1,2^, Achim Aigner ^4^, Chiara Andolfi ^5^, Florian Handle ^5^, Stefanie Rheinheimer ^6^, Ulrike Fischer ^6^, Uta D. Immel ^7^, Verena Lieb^1,2^, Eckart Meese ^6^, Zoran Culig ^5^, Bernd Wullich ^1,2^, Helge Taubert ^1,2,*,†^ and Sven Wach ^1,2,†^


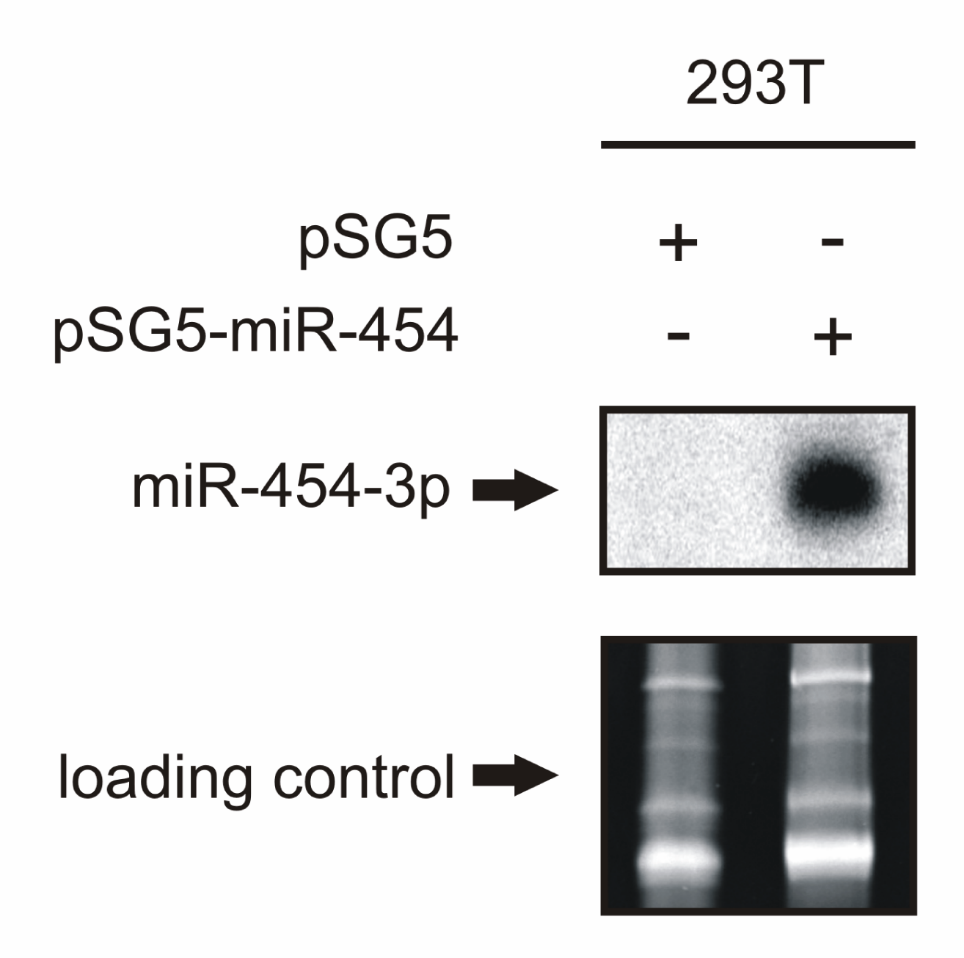


**Suppl. Fig. S1**: Northern blotting with radiolabeled DNA probes specific for miR-454-3p

HEK-293T cells were transfected with an empty expression plasmid pSG5 (left lane) or an expression plasmid pSG5 containing the miR-454 sequence (right lane). After hybridization of the RNA with radiolabeled DNA probe specific for miR-454-3p a signal for miR-454-3p could be detected in the right lane representing the plasmid pSG5-miR-454 but not in the left lane with the empty pSG5 plasmid.


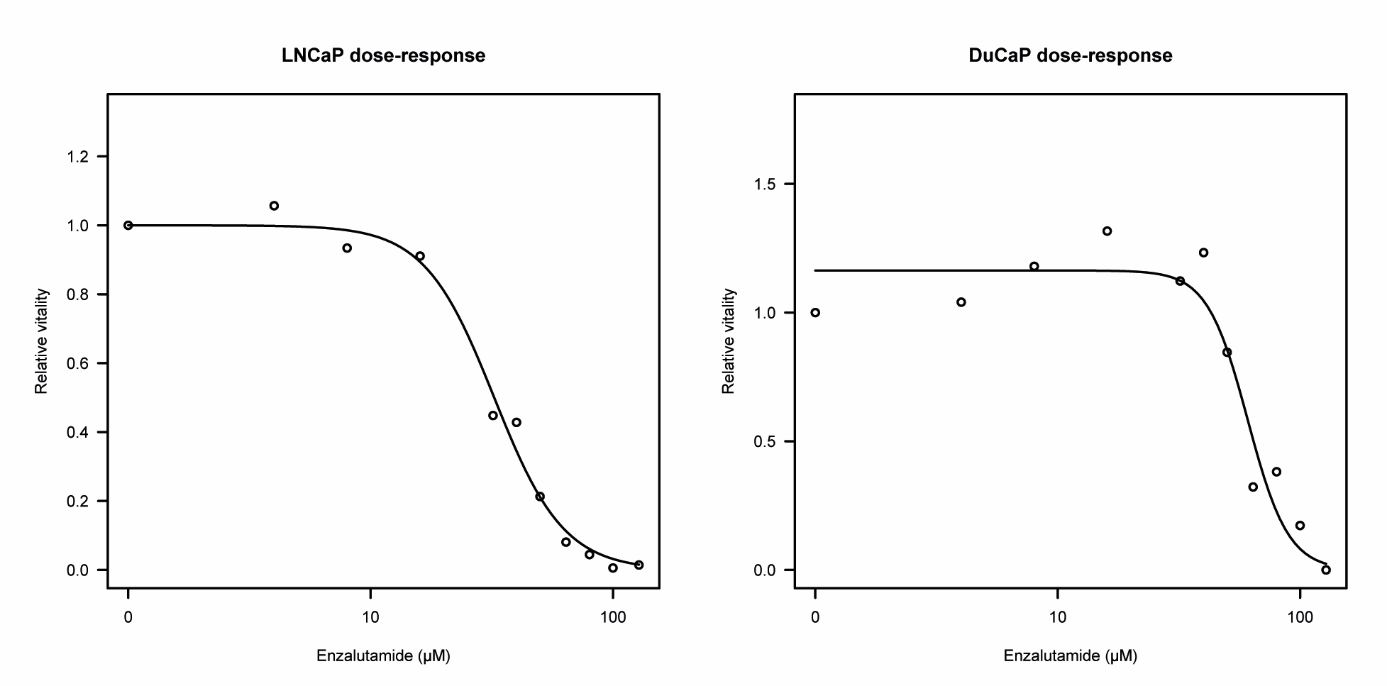


**Suppl. Fig. S2**: Enzalutamide dose response curves for LNCaP Abl EnzR und DuCaP EnzR cells

The LNCaP Abl EnzR cell line showed a half effective doses (ED50) for enzalutamide of 32.4µM (28.5-36.2µM) and the DuCaP EnzR cell line revealed an ED50 of 60.8µM (52.1-69.6µM).


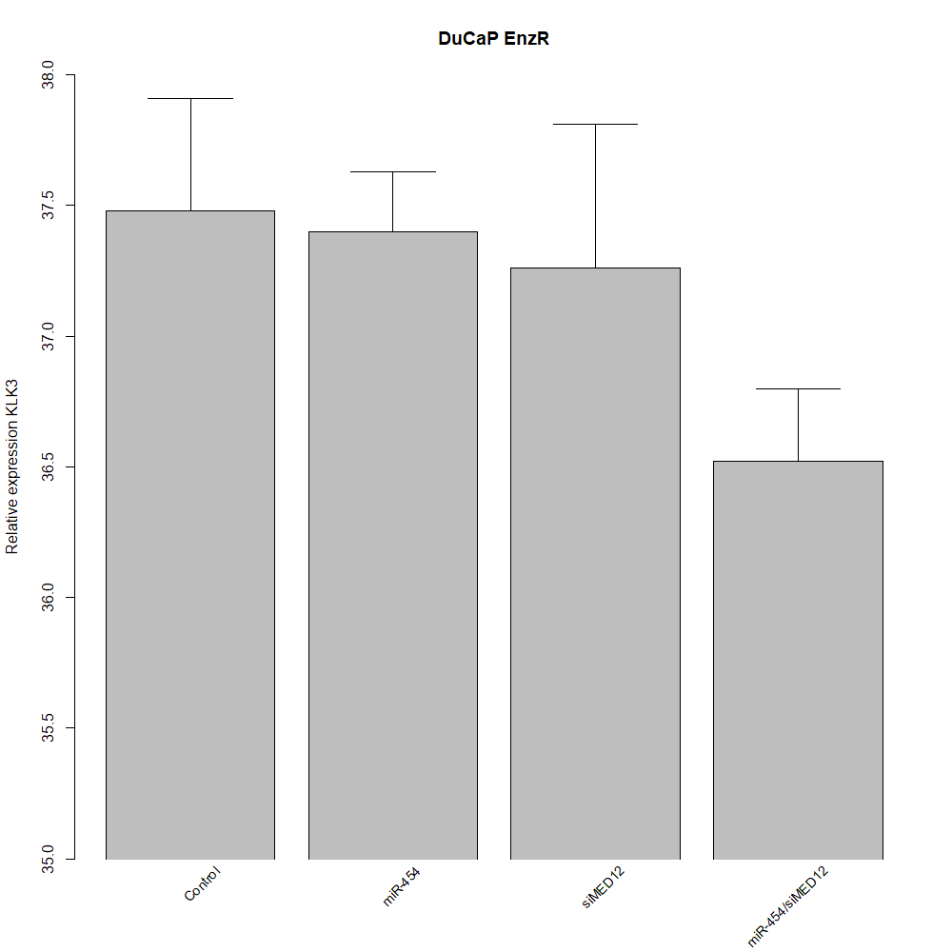


| **Treatments** | **diff** | **lwr** | **upr** | **p adj** |
| --- | --- | --- | --- | --- |
| **miR-454-Control** | -0.08 | -1.1030277 | 0.9430277 | 0.9940435 |
| **miR-454/siMED12-Control** | -0.9633333 | -1.9863610 | 0.05969437 | 0.0650743 |
| **siMED12-Control** | -0.22 | -1.2430277 | 0.8030277 | 0.8985959 |
| **miR-454/siMED12-miR-454** | -0.8833333 | -1.9063610 | 0.13969437 | 0.092685 |
| **siMED12-miR-454** | -0.14 | -1.1630277 | 0.8830277 | 0.970081 |
| **siMED12-miR-454/siMED12** | 0.7433333 | -0.2796944 | 176.636.104 | 0.1707228 |

**Suppl. Fig. S3A**: Gene expression of the AR-target gene KLK3 in the DuCaP EnzR after different
 treatments

KLK3 mRNA expression was measured by qRT-PCR. Relative KLK3 expression is shown as 40-dCt of KLK3-GAPDH at different treatments. The Anova post hoc Tukey test (pairwise comparison) did not reveal significant differences for KLK3 mRNA expression after treatments compared with the control (p adjusted > 0.05).


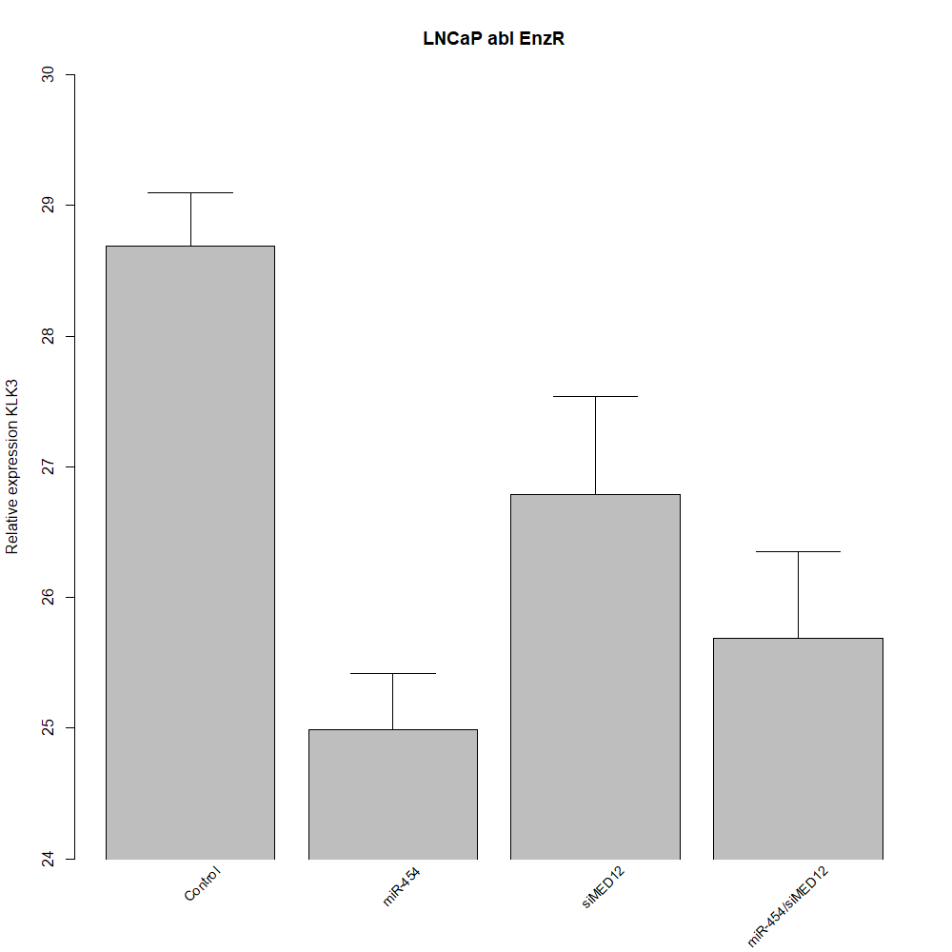


| **Treatments** | **diff** | **Lwr** | **upr** | **p adj** |
| --- | --- | --- | --- | --- |
| **miR-454-Control** | -3.7033333 | -5.2213668 | -2.1852999 | **0.0002382** |
| **miR-454/siMED12-Control** | -3.0000000 | -4.5180335 | -1.4819665 | **0.0010207** |
| **siMED12-Control** | -1.8966667 | -3.4147001 | -0.3786332 | **0.0166291** |
| **miR-454/siMED12-miR-454** | 0.7033333 | -0.8147001 | 2.2213668 | 0.4884908 |
| **siMED12-miR-454** | 1.8066667 | 0.2886332 | 3.3247001 | **0.0214912** |
| **siMED12-miR-454/siMED12** | 1.1033333 | -0.4147001 | 2.6213668 | 0.1705612 |

Significant values are in bold face.

**Suppl. Fig. S3B**: Gene expression of the AR-target gene KLK3 in the LNCaP Abl EnzR after different
 treatments

KLK3 mRNA expression was measured by qRT-PCR. Relative KLK3 expression is shown as 40-dCt of KLK3-GAPDH at different treatments. The Anova post hoc Tukey test (pairwise comparison) revealed significant differences for KLK3 mRNA expression after all treatments compared to the control, i.e for treatment with miR-454 (p=0.0002382), siMED12 (p=0.0166291), combination miR-454/siMED12 (p=0.0010207) and between the treatments with miR-454 vs. siMED12 (p=0.0214912).


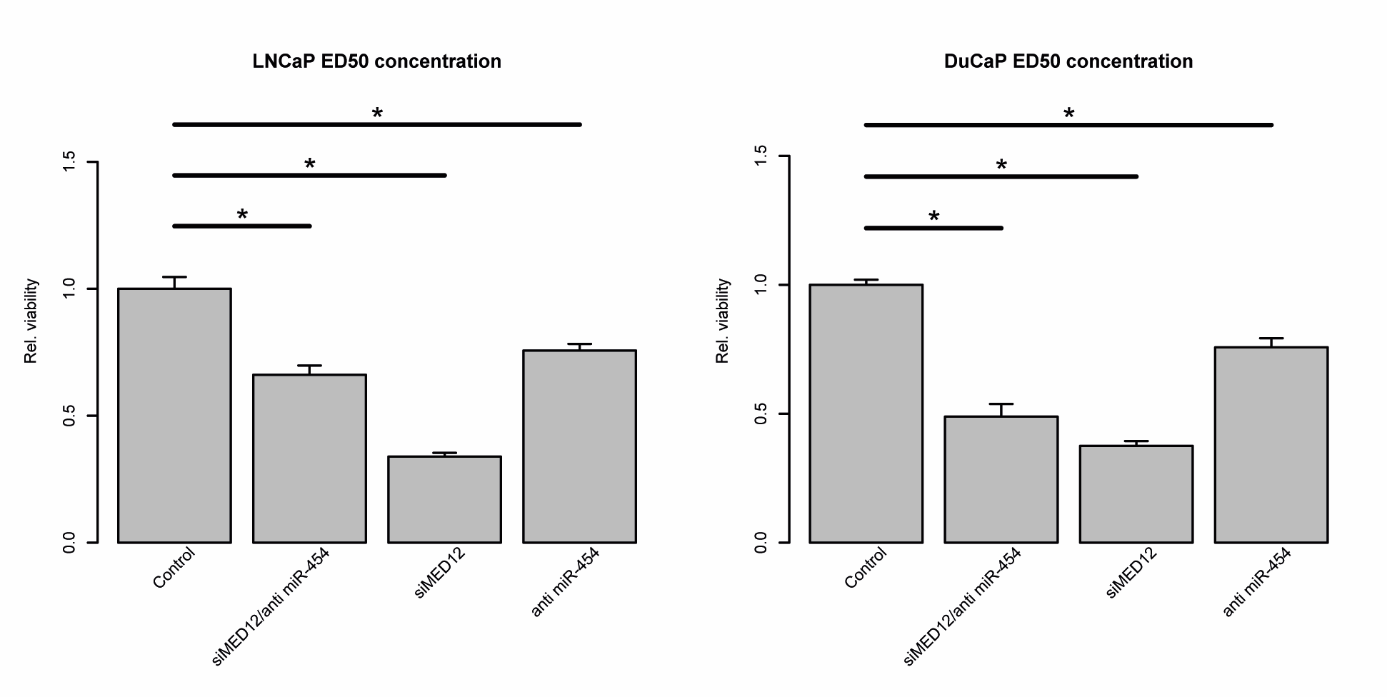


**Suppl. Fig. S4**: Relative viability of LNCaP Abl EnzR and DuCaP EnzR cells kept under ED50 enzalutamide concentrations transfected with siMED12, anti-miR-454-3p or combined siMED12/anti-miR-454-3p

Compared to the controls in both cell lines transfection with siMED12, anti-miR-454-3p or combined siMED12/anti-miR-454-3p resulted in a significant reduction in cell viability.
